# A novel technique of spacer implantation and proton beam therapy for hilar cholangiocarcinoma: a case report

**DOI:** 10.3389/fonc.2026.1747997

**Published:** 2026-02-27

**Authors:** Wencui Yang, Junetsu Mizoe, Xiaolong Wang

**Affiliations:** 1 Department of Radiation Oncology, Xi’an International Medical Center Hospital, Xi’an, China; 2 Sapporo High Functioning Radiotherapy Center, Sapporo Kojinkai Memorial Hospital, Sapporo, Japan; 3 Department of Thyroid and breast surgery, Xi’an International Medical Center Hospital, Xi’an, China

**Keywords:** bioabsorbable spacer, case report, hilar cholangiocarcinoma, proton beam therapy, surgical spacer placement

## Abstract

The primary treatment for hilar cholangiocarcinoma is a surgical resection. However, most patients have already lost the opportunity for surgical treatment at the time of the initial diagnosis. Photon therapy (X-ray) has limited efficacy, whereas proton beam therapy (PBT) can improve local control. However, when tumors are located near the gastrointestinal tract, the therapeutic effect remains limited. Space-making particle therapy (SMPT) has emerged as a strategy to address the clinical challenge of high-dose radiation exposure to nearby organs at risk (OARs). This technique involves the surgical implantation of a bioabsorbable spacer prior to particle therapy delivery. Therefore, we are interested in the use of SMPT to preserve the gastrointestinal tract. A 77-year-old man with hilar cholangiocarcinoma underwent placement of a bioabsorbable spacer and then received radical PBT of 72.6 Gy (relative biological effectiveness, RBE) in 22 fractions. Surgery was not included in the original treatment plan. However, approximately 5 months after completing PBT, the patient developed biliary fluid accumulation, which subsequently required surgical intervention. Notably, pathological examination of the resected irradiated field revealed a complete pathological response (pCR), providing direct evidence of the treatment efficacy. This report describes one of the first cases in which a bioabsorbable spacer was surgically placed to preserve the gastrointestinal tract during PBT. SMPT is an innovative solution for hilar cholangiocarcinoma adjacent to the gastrointestinal tract. This case demonstrated the early feasibility and short-term efficacy of SMPT, but long-term follow-up is needed.

## Introduction

Cholangiocarcinoma is a heterogeneous malignant tumor that originates in the biliary epithelium. Hilar cholangiocarcinoma (also known as Klatskin tumor) occurs at the confluence of the left and right hepatic ducts, accounting for approximately 50%–60% of all cholangiocarcinoma cases (1, 2). Intrahepatic and hilar cholangiocarcinomas, including advanced unresectable lesions, are associated with a poor prognosis, with a 5-year survival rate of 5%–10% (3). Complete surgical resection is considered the only treatment for long-term survival (4); however, many patients present at an advanced stage and are ineligible for surgery because of vascular invasion, poor performance status, or distant metastasis. Radiotherapy plays an important role in treating unresectable cholangiocarcinoma. However, the efficacy of X-rays is limited by the low radiation tolerance of adjacent OARs, notably the stomach, duodenum, and small intestine. To respect their dose constraints, compromises in target volume coverage or prescribed dose intensity are often necessary, which may subsequently compromise long-term local control and overall survival (OS).

Proton beam therapy (PBT) offers superior physical and biological advantages (approximately 1.1 times) than X-ray due to the “Bragg peak.” It reduces the adverse effects on the normal liver and small intestine owing to the Bragg peak (5, 6). Recent clinical studies have indicated that PBT can achieve 5-year local control rates of 80%–90% (7, 8). However, PBT involves uncertainties, particularly regarding the distal high-dose region, leading to severe side effects such as intestinal adhesions, gastrointestinal bleeding, and perforation (9).

Gastrointestinal toxicity following particle therapy is a recognized poor prognostic factor for patients with cancer. Consequently, dose reduction is often mandated to mitigate this risk, a compromise that may inadvertently elevate the risk of local recurrence (10). To overcome this dosimetric challenge, the medical community has proposed space-making particle therapy (SMPT). This innovative approach involves inserting a biodegradable spacer material between the tumor and gastrointestinal tract, effectively creating a protective distance that facilitates significant dose escalation to the target (11). This report details the successful application of the SMPT in a patient with refractory hilar cholangiocarcinoma. A bioabsorbable polyglycolic acid spacer (Neskeep^®^, Alfresa Pharma Corporation, Osaka, Japan) was used to enable the delivery of curative proton radiation doses.

## Case presentation

**Figure 1:**
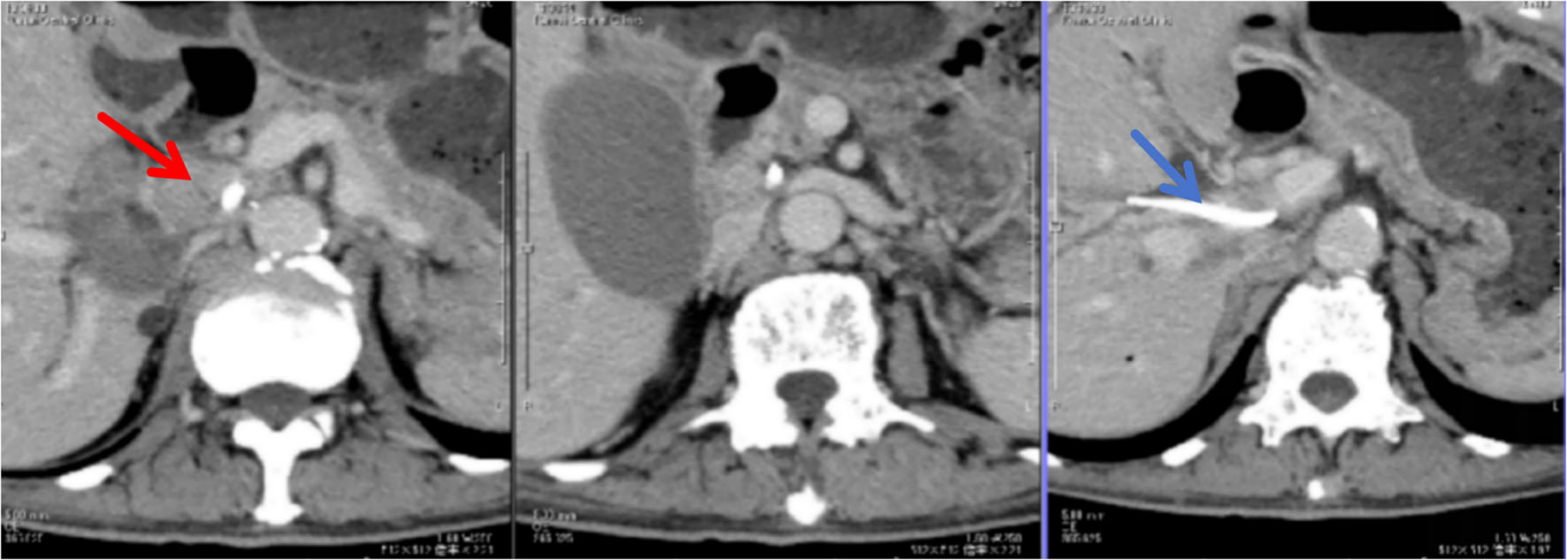
An abdominal computed tomography (CT) scan obtained following neoadjuvant chemotherapy, revealing a tumor located at the hepatic portal (indicated by a red arrow) and an indwelling biliary stent (indicated by a blue arrow).

A 77-year-old man presented with complaints of hilar cholangiocarcinoma for the past 6 months and chemotherapy for the past 1 month. He was initially diagnosed with hilar cholangiocarcinoma at a local hospital. Abdominal computed tomography (CT) scan (09–12–2021) revealed a space-occupying lesion in the hepatic hilum, accompanied by intrahepatic biliary dilatation and hydrops of the gallbladder (**Figure 1**).

**Figure 2:**
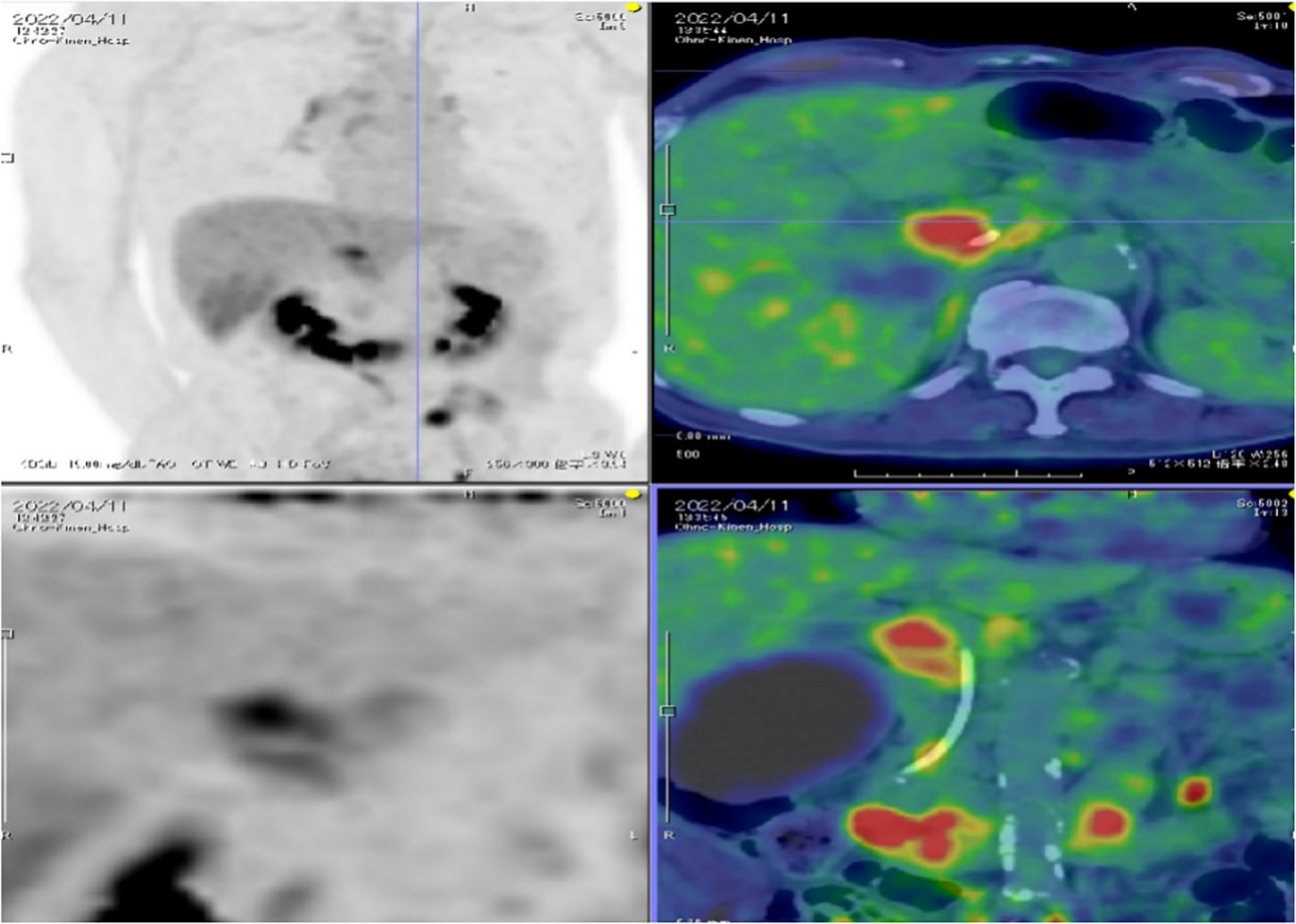
The patient’s positron emission tomography-computed tomography (PET-CT) performed prior to proton therapy, exhibiting a hypermetabolic focus at the hepatic portal with associated retroperitoneal lymph node metastasis, as well as the indwelling biliary stent.

Endoscopic retrograde cholangiopancreatography (ERCP) with biopsy was performed, and a biliary stent was placed. Biopsy pathology confirmed hilar cholangiocarcinoma. Therefore, the definitive pathological diagnosis was hilar cholangiocarcinoma. The TNM staging was cT2aN1M0, Stage III. The patient received two cycles of gemcitabine and cisplatin chemotherapy on 14-12-2021 and 14-01-2022 at a local hospital. However, after two cycles of chemotherapy, follow-up positron emission tomography (PET)-CT still demonstrated a space-occupying lesion in the hepatic hilum, with a maximum standardized uptake value (SUV max) of 6.8. Associated findings included intrahepatic biliary dilatation and hydrops of the gallbladder (**Figure 2**). After the completion of two cycles of chemotherapy, re-assessment revealed stable disease (SD). He was admitted to the radiotherapy department for further management. The patient had no history of smoking or alcohol consumption and denied any family history of cancer.

Multidisciplinary treatment (MDT) discussion concluded that this patient was unsuitable for surgery due to his poor performance status and personal refusal, despite having only SD after two cycles of chemotherapy with a residual 3 cm tumor. As a result, radical PBT was pursued for optimal local control of the disease. The managing physician recommended referral for local control with palliative or radical radiotherapy. As we all know, the radiotherapy usually includes X-ray and PBT. The patient presented to our department for PBT. The tumor location was next to the hepatic hilum, its histology (cholangiocarcinoma, typically of low radiosensitivity), and its proximity to the duodenum and small intestine posed significant challenges for PBT. The inevitable small intestinal dose constraints would severely limit the radiation dose to the tumor, precluding the delivery of a tumoricidal dose. To enhance target dose coverage while reducing the dose to normal tissues, a bioabsorbable spacer (Neskeep^®^) was implanted between the tumor and the intestines before delivering PBT.

**Figure 3:**
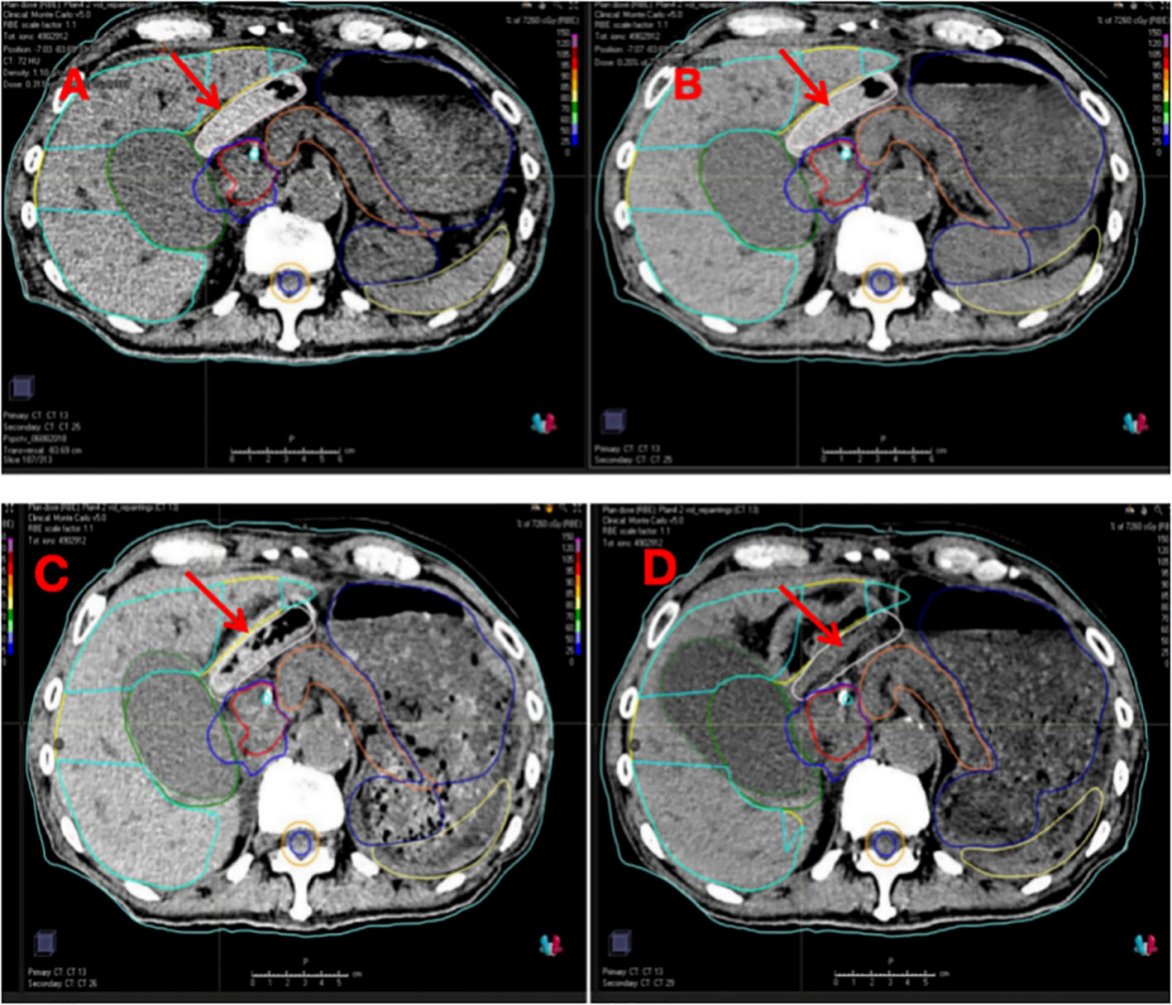
**(A)** The target volume delineated on the simulation CT, with **(B–D)** illustrating the target volumes on the CT scans from the 1st, 11th, and 20th treatment fractions, respectively. The red arrows indicated the position of the PGA.

On 11 May 2022, the patient underwent open laparotomy under general anesthesia for Neskeep^®^ implantation. The absorbable spacer was placed between the tumor and intestines/liver and fixed to the greater omentum with nonabsorbable sutures to prevent displacement. The postoperative recovery was uneventful. The proton treatment machine at our institution was delivered by IBA Proteus^®^ONE. It features an isochronous synchrocyclotron that generates a proton beam, which is then meticulously shaped and directed by a 223° partial rotation compact gantry and spot-scanning beam delivery nozzle. The planning system used at our institution was RayStation version 10A. CT simulation for PBT was performed on 18 May 2022. During the simulation and daily PBT, the patients were immobilized in the supine position using a blue vacuum pad with their arms raised above their heads. In treatment planning, a 16-row multidetector CT scanner (Siemens) was used to acquire 1-mm-thick images. A conventional CT scan was first acquired, followed by contrast-enhanced CT and four-dimensional CT (4D-CT). The gross tumor volume (GTV) included the hilar lesion and positive lymph nodes visible on CT and PET-CT. The internal gross tumor volume (iGTV) was delineated using 4D-CT. The clinical target volume (CTV) was defined as the iGTV with a 5 mm margin. The prescribed dose was 72.6 Gy (RBE), delivered in 22 fractions. In the dose-volume analysis, the duodenum received a D2cc of 25.5 Gy (RBE) and a maximum dose of 53.2 Gy (RBE). The gallbladder received a D2cc of 66.12 Gy (RBE) and a maximum dose of 73.2 Gy (RBE). PBT was administered from Monday to Friday. The treatment was performed without interruption. Owing to the frail condition of the patient’s, concurrent chemotherapy was not administered. During treatment, the patient reported occasional fatigue but denied nausea, vomiting, abdominal distension, and pain. X-ray image-guided registration was performed before each fractionation. Diagnostic CT scans were performed weekly during treatment and fused with the planning CT to assess tumor changes and the status of the Neskeep^®^ spacer (**Figure 3**). Five CT registrations were performed during treatment, showing no significant shrinkage or displacement of the Neskeep^®^ spacer. **Figure 3** shows the planning CT and treatment CT images from fractions 1, 11, and 20, demonstrating slight tumor shrinkage and minor internal absorption of the spacer but no displacement or significant volume change. The CTV coverage remained within a 10% variation range; therefore, the treatment plan was not modified.

**Figure 4:**
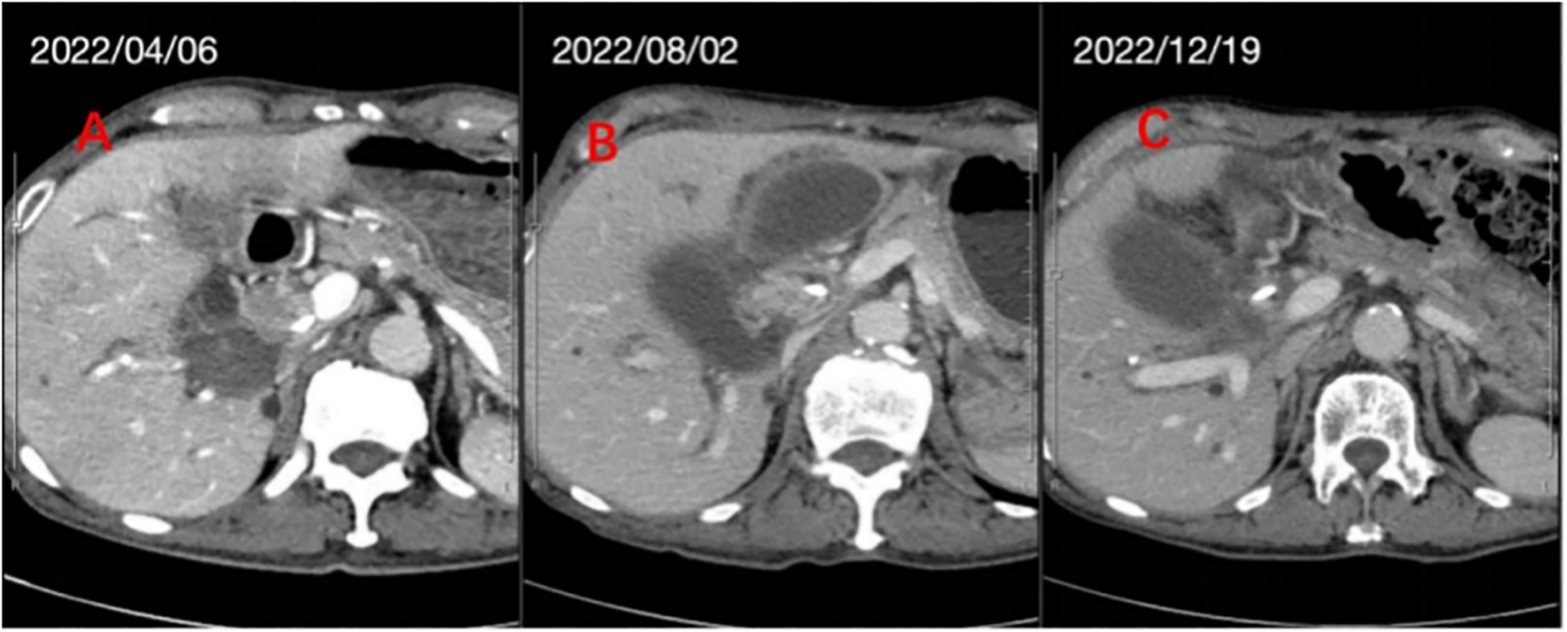
CT images before treatment **(A)**, and at 1 month **(B)** and 5 months **(C)** after the proton therapy.

Follow-up abdominal contrast-enhanced CT performed one month after treatment completion showed significant shrinkage of the lesion (**Figure 4**). The tumor marker CA19–9 demonstrated a marked decline, dropping from a pretreatment level of 57,662 U/mL (04-07-2022) to 66.8 U/mL (19-12-2022) following the initiation of therapy. In the 4 months since PBT, the patient underwent surgery for biliary fluid accumulation. Postoperative pathology indicated complete remission of the tumor. Follow-up every 3 months for the next 2 years showed a complete response on abdominal CT.

## Discussion

Hilar cholangiocarcinoma has a relatively low incidence and poor prognosis among biliary tract cancers (12, 13). The primary treatment for hilar cholangiocarcinoma is surgical resection. Other treatment modalities include postoperative radiotherapy, chemotherapy, and targeted therapy. However, many patients present with locally advanced disease at initial diagnosis, precluding curative resection, or have high-risk factors after radical surgery, requiring adjuvant radiotherapy. Owing to the proximity of hilar cholangiocarcinoma to OARs, such as the duodenum and small intestine, the radiation dose to the tumor region is often limited by OARs constraints. Studies have shown a significant correlation between radiation dose and local control rates, as well as long-term survival in cholangiocarcinoma (14). Increasing the dose for these cancers can improve local control by approximately 20%–40%. Therefore, addressing the challenge of tumor dose escalation remains a difficult and active area of clinical research.

SMPT is an emerging innovative concept whose core strategy involves implanting a spacer to create separation between the tumor and adjacent OARs (15). The rapidly growing number of recent publications demonstrates the therapeutic efficacy of SMPT in various malignancies and its potential to expand treatment indications (15–17). SMPT using expanded polytetrafluoroethylene (ePTFE) spacers for hepatocellular carcinoma (HCC) has been previously reported (15–18). However, owing to the non-absorbable nature of ePTFE, long-term follow-up has identified serious adverse events, notably gastrointestinal perforation (17). A novel bioabsorbable spacer (Neskeep^®^) has been applied to abdominal tumors, such as pancreatic cancer, cholangiocarcinoma, and liver cancer (2). These PGA spacers measured 200 mm in length and 100 mm in width and were available in three thicknesses (5 mm, 10 mm, and 15 mm). After implantation, they are absorbed by the body via hydrolysis. In a clinical trial, Sasaki et al. (19) reported the safety and efficacy of PGA spacer implantation before proton therapy.

Therefore, with the development and clinical approval of PGA spacers, our hospital used a PGA spacer for SMPT in this case of hilar cholangiocarcinoma. The advantages of PGA spacers include their absorbable nature and ability to customize their thickness based on tumor morphology and anatomical complexity (19). Previous studies on other malignancies have confirmed the effectiveness of PGA spacers in SMPT (20, 21).

Typically, the implantation of a PGA spacer requires a MDT discussion involving radiation oncology, surgery, internal medicine, and radiology. The general eligibility criteria included: ① an expected survival of >6 months; ② an Eastern Cooperative Oncology Group (ECOG) performance status of 0–2; ③ well-defined tumor borders without significant adhesion to organs at risk (OARs); and ④ favorable anatomy to facilitate surgical manipulation.

The MDT concluded that the patient was unsuitable for surgery due to his poor performance status and personal preference. However, the patient met the eligibility criteria for combined spacer implantation. Although the patient presented with retroperitoneal lymph node metastasis, it was a solitary lesion (oligometastasis) located close to the primary tumor site. This overall disease pattern was thus more consistent with locoregional progression rather than with widespread dissemination. Considering the patient’s compromised performance status and strong motivation for aggressive treatment, the MDT formulated a comprehensive curative-intent strategy. As part of this approach, radical radiotherapy (72.6 Gy RBE) was planned for the primary tumor and the adjacent lymph node, with the aim of achieving maximal locoregional control and pursuing the possibility of long-term survival.

The tumor borders were well-defined, without significant adhesions to the duodenum or small intestine. Given these favorable conditions, the estimated success rate of the Neskeep^®^ implant procedure exceeded 90%, fully meeting the established indications. On 11 May 2022, the patient underwent Neskeep^®^ implantation under general anesthesia. The spacer was fixed to the greater omentum using nonabsorbable sutures to prevent displacement or migration. This patient was the first Neskeep^®^ implantation case at our hospital. The patient successfully completed proton therapy. Weekly CT scans during treatment showed no displacement or migration of the Neskeep^®^ spacer (**Figure 3**). One month after PBT, the tumor marker CA19–9 showed a significant decrease. Abdominal contrast-enhanced CT revealed marked tumor shrinkage. Five months after PBT, the tumor marker CA19-9 was normal, and abdominal contrast-enhanced CT revealed that the tumor was in complete remission. A critical juncture in the management of this case warrants emphasis: the initial treatment plan was radical radiotherapy, with surgery not being an intended component of the treatment. Subsequent emergency surgery was performed due to an unexpected complication, acute gallbladder perforation, during which the hilar region encompassing the planned radiotherapy target volume was resected. Unexpectedly, postoperative pathology revealed a pathological complete response (pCR). Therefore, the following factors must be comprehensively considered when interpreting this pCR finding. First, the physical and biological effects of PBT should be thoroughly evaluated. Owing to its superior target dose conformity and steep dose fall-off gradient. Second, we delivered a radical dose to the tumor because of the PGA spacer. Third, the patient completed two cycles of systemic chemotherapy before proton therapy. Chemotherapy may not only eradicate potential micrometastases but also exert direct cytotoxic or radiosensitizing effects on the local tumor, thereby synergizing with the subsequent proton therapy to collectively enhance tumoricidal activity in the primary region. Abdominal CT performed prior to proton therapy in this patient revealed biliary duct dilatation and significant gallbladder hydrops. Given that the site of fluid accumulation was adjacent to the target area and considering the occurrence of gallbladder perforation 5 months after proton therapy, the effect of high-dose proton irradiation cannot be ruled out. Other contributing factors include tumor treatment response and the mechanical effect of the implanted spacer. Subsequent follow-up imaging showed no enhancement in the fluid accumulation area, and the region remained stable or even gradually shrank. Furthermore, a comprehensive systemic evaluation revealed no new lesions, thereby largely excluding tumor progression as the cause. This finding suggests that during spacer candidate selection, factors such as the gallbladder and biliary ducts should be considered to avoid similar complications in the future.

A 40% PGA absorption rate was observed. Incomplete absorption was considered for two reasons: first, insufficient time may not have elapsed for full absorption. On the other hand, bile leakage from gallbladder perforation could have altered the potential of hydrogen (pH) of the PGA, which adversely affected the material’s properties and absorption. This report describes one of the first cases of surgical placement of a bioabsorbable spacer to preserve the gastrointestinal tract during PBT at our hospital. SMPT is an innovative solution for hilar cholangiocarcinoma adjacent to gastrointestinal tract. Serial follow-up examinations conducted at 3-month intervals confirmed a complete response on the abdominal CT.

Before 2020, most institutions employed open laparotomy for PGA implantation. Since then, laparoscopic implantation has been gradually adopted, further shortening the waiting time for treatment and improving patient compliance. For the absorbable PGA spacer, fixation to the greater omentum with nonabsorbable sutures is used to prevent displacement, and clinical practice has shown this method to be highly feasible with stable efficacy. The SMPT technique, utilizing a PGA spacer, provided a safer and more suitable treatment option for this complex case. Such a complete clinical response is relatively rare in patients with hilar cholangiocarcinoma treated with radiotherapy alone. The outcome in this case may reflect the significant tumoricidal effect of high-dose proton therapy, facilitated by the PGA spacer.

## Conclusion

PGA (Neskeep^®^)-assisted proton therapy is a feasible and promising treatment option for unresectable hilar cholangiocarcinoma. However, strict patient selection and long-term follow-up are necessary to fully evaluate its efficacy and to monitor potential complications.

## Data Availability

The datasets presented in this study can be found in online repositories. The names of the repository/repositories and accession number(s) can be found in the article/supplementary material.
